# Histopathological and Immunohistochemical Characteristics of Different Types of Cardiac Amyloidosis

**DOI:** 10.3390/ijms251910667

**Published:** 2024-10-03

**Authors:** Zarina V. Gioeva, Liudmila M. Mikhaleva, Nikita A. Gutyrchik, Alexey V. Volkov, Mikhail A. Popov, Nikolay K. Shakhpazyan, Valentina V. Pechnikova, Konstantin Y. Midiber, Elena V. Reznik, Lev V. Kakturskij

**Affiliations:** 1 Avtsyn Research Institute of Human Morphology, Petrovsky National Research Centre of Surgery, 117418 Moscow, Russia; mikhalevalm@yandex.ru (L.M.M.); gyt94@yandex.ru (N.A.G.); alex.volkoff@gmail.com (A.V.V.); nshakhpazyan@gmail.com (N.K.S.); valiagtx@yandex.ru (V.V.P.); midiberkonst@gmail.com (K.Y.M.); levkaktur@mail.ru (L.V.K.); 2 Institute of Medicine, Peoples’ Friendship University of Russia named after Patrice Lumumba, 6 Miklukho-Maklaya St., 117198 Moscow, Russia; 3 Department of Cardiac Surgery in M. F. Vladimirskiy Moscow Regional Research Clinical Institute, 129110 Moscow, Russia; popovcardio88@mail.ru; 4 Department of Internal Medicine №2, Pirogov Russian National Research Medical University, 117997 Moscow, Russia; elenaresnik@gmail.com

**Keywords:** amyloid, cardiac amyloidosis, endomyocardial biopsy, transthyretin, immunoglobulin light chain, immunohistochemistry

## Abstract

Cardiac involvement is the most important factor determining prognosis in patients with systemic amyloidosis. This retrospective observational study of 98 patients with amyloidosis was undertaken to assess the amyloid types that are most likely to affect the heart, describe histopathological and clinical features of cardiac amyloidosis, and estimate the number of cases not diagnosed clinically prior to death. All cases were divided into two groups based on the method of examination. The first group included 46 patients with cardiac amyloidosis revealed via endomyocardial biopsies (EMBs), and the second group included 52 amyloidosis patients who did not undergo EMBs, in whom cardiac involvement was identified only at autopsy. The EMBs demonstrated that AL amyloidosis was detected in 21 (46%) specimens, ATTR amyloid in 24 cases (52%), and AA amyloid in 1 case (2%). The autopsy reports defined 15 (46%) cases of AL amyloidosis, 21 (40%) of ATTR and 16 (31%) of AA amyloidosis. It should be noted that a clinical diagnosis of ATTR amyloidosis was made only in 9.5% of patients from the autopsy group, suggesting that ATTR may be an underdiagnosed cause of heart failure in elderly patients. The most intense amyloid deposits were determined in biopsy and autopsy specimens of patients with AL kappa amyloidosis, underlying a poorer prognosis.

## 1. Introduction

The heart is one of the predominant organs affected by systemic amyloidosis. Currently, ten different amyloidogenic precursor proteins are known that can affect the heart; nine of them are responsible for systemic amyloidosis, and one is responsible for localized amyloidosis [1].

The most common type of amyloidosis causing amyloid cardiomyopathy is AL amyloidosis, which is considered a variant of lymphoplasmacytic dyscrasia related to the proliferation of abnormal plasma- or B-cell clones in the bone marrow [2,3]. It is associated with the overproduction of monoclonal-free immunoglobulin light chains that are capable of inducing amyloidogenesis [4,5,6]. AL amyloidosis is a systemic rapidly progressive disease that most often occurs in people over the age of 50. It may develop as a nosological unit or co-occur with multiple myeloma in 7–15% of patients [7,8]. Heart involvement is reported in 50–80% of cases of this amyloidosis type [9,10,11].

Transthyretin amyloidosis (ATTR) is the second most common cause of heart involvement in amyloidosis. The precursor protein of ATTR is transthyretin, which is normally produced in the liver and transports thyroxine and retinol [12,13]. There are two different types of ATTR amyloidosis: wild-type ATTR (ATTRwt) and hereditary (ATTRv) amyloidosis [14,15].

ATTRwt is characterized by high cardiotropism, and almost 100% of patients show signs of cardiac involvement. In most cases, previously undiagnosed ATTR-wt is uncovered as an incidental finding at the post-mortem examination of the heart in 25–40% of individuals over the age of 80 years, and in 32% of patients older than 75 years who have heart failure with preserved ejection fraction [16].

ATTRv amyloidosis is caused by a mutation in the transthyretin gene and is characterized by heart involvement in 30–100% of cases. So far, over 140 different mutations associated with ATTRv have been described that can cause various phenotypic manifestations affecting individual organs, including the heart and nerves (polyneuropathy with autonomic dysfunction), or give rise to a mixed clinical phenotype [17].

The precursor protein of AA amyloidosis is serum amyloid A (SAA). It is mainly produced in the liver by proinflammatory stimuli such as IL-6, IL-1, and TNFα in response to inflammation and can contribute to tumor development [18]. Cardiac involvement in this type of amyloidosis is considered rare. Also, apolipoprotein A-I, II and IV amyloidosis, fibrinogen alpha chain and gelsolin amyloidosis have been reported [19,20,21]. Cardiac involvement occurs in patients with hereditary systemic amyloidosis where the protein β2-microglobulin variant is the causing agent [22]. Isolated atrial amyloidosis (AANF) caused by the accumulation of atrial natriuretic peptide has been detected more frequently over the past several years [23].

The prognosis in patients with amyloidosis is variable and often depends on cardiac involvement. Cardiac amyloidosis is the cause of death in approximately 61% of the patients [24]. As reported in the literature, cardiac amyloidosis is considered a cause of nearly 10% of all non-ischemic cardiomyopathies [7].

The major problem with recognizing cardiac amyloidosis in its early stages is its ability to mimic other disorders [25]. Later clinical manifestations are determined by the involvement of myocardium, endocardium, pericardium, and large and small coronary arteries, and represented by restrictive cardiomyopathy, systolic heart failure, orthostatic hypotension and cardiac conduction system disorders. Frequently, these syndromes co-exist [26,27].

This study was undertaken to analyze the percentage of the most common types of amyloidosis revealed in two patient groups. The first group consisted of patients with cardiac amyloidosis detected via EMB during life. The second group included patients in whom postmortem histological examination was performed to determine the number of cardiac amyloidosis cases not diagnosed clinically prior to death.

## 2. Results

### 2.1. Endomyocardial Biopsy Findings

#### 2.1.1. Histology and Immunohistochemistry

In this study, 46 EMB specimens from 28 males (61%) and 18 females (39%) aged 45–91 years (mean 71 years) were evaluated.

**Figure 1:**
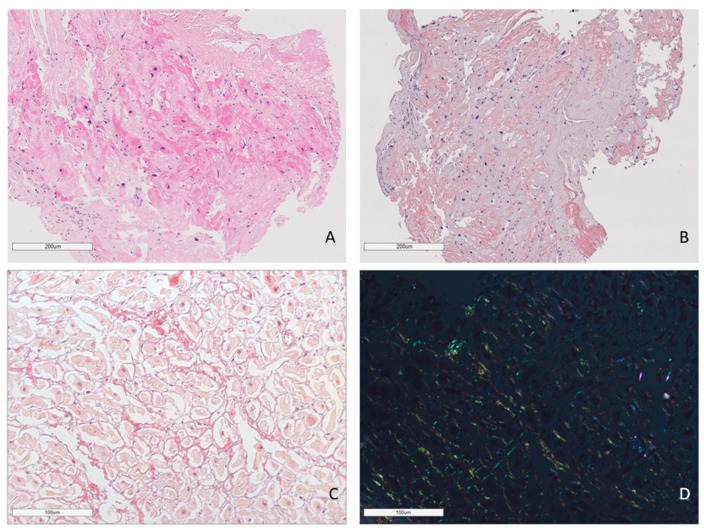
Amyloid deposits in the EMB sample. AL lambda amyloidosis. Homogenous eosinophilic deposits of amyloid after H&E staining (**A**) that demonstrate bright red color after Congo red staining (**B**). ×100. AL kappa amyloidosis. Diffuse pericellular and intravascular deposition of amyloid (**C**) displaying apple-green birefringence under polarized light (**D**). Staining with Congo red, ×200.

In all cases, amyloid deposits were identified with hematoxylin and eosin (H&E) stains as homogenous eosinophilic material (homogenous sties of bright pink color). Amyloid deposits also produced a characteristic apple-green birefringence on the black background under polarized light after Congo red (CR) staining (Figure 1).

Based on the immunohistochemistry (IHC) typing results, the diagnosis of AL amyloidosis was established in 21 (46%) patients; 6 of them (29%) had the kappa type of light chain amyloidosis (AL kappa) and 15 (71%) had the lambda type of light chain amyloidosis (AL lambda). ATTR was found in 6 women (25%) and 18 men (75%). Based on the EMB results, AA amyloidosis was diagnosed only in one patient. The correlation analysis between patient age and amyloid type in EMB specimens revealed a peak level of AL amyloid type in people aged 70 years or older (48%), while ATTR amyloidosis demonstrated two peaks—during the eighth (54%) and ninth (30%) decades of life (Table 1).

**Figure 2:**
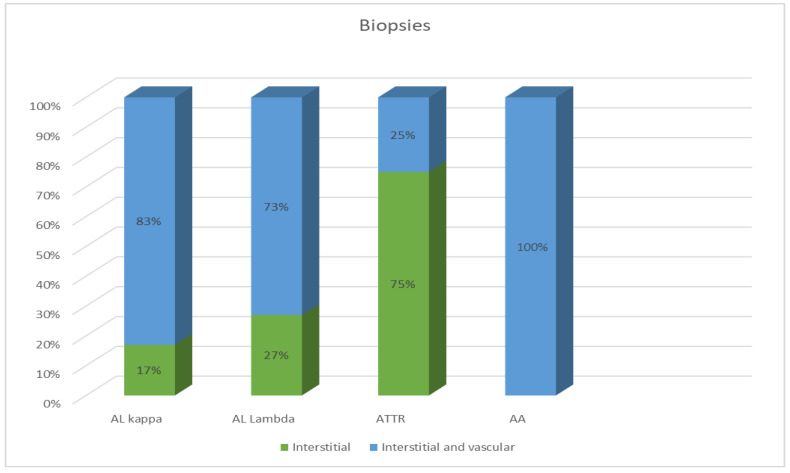
Comparative distribution of amyloid deposits in the EMB specimens of patients with different types of amyloidosis.

Most EMBs from patients with AL amyloidosis were characterized by a reticular or pericellular pattern of amyloid deposition. In 83% of the AL kappa amyloidosis cases, amyloid deposits were distributed both in the interstitial and intravascular tissues, and in 17% of cases, they were distributed only in the interstitial tissue. Among the AL lambda amyloidosis cases, both interstitial and vascular amyloid deposits were observed in 73% of cases, while an interstitial pattern was found in 27%. In most specimens, ATTR amyloidosis was characterized by the presence of clumpy multifocal amyloid deposits in the myocardial stroma. Interstitial focal amyloid depositions were found in 75% of the examined specimens, and both vascular and interstitial depositions were found in 25%. A single detected case of AA amyloidosis was characterized by moderate amyloid deposits both in the stroma and blood vessel walls (Figure 2).

The largest affected areas were found in patients with AL kappa amyloidosis, where a pericellular pattern of amyloid deposition predominated, and cardiomyocytes adjacent to the amyloid deposits were often atrophic.

#### 2.1.2. Clinical Information

In our study, the clinical diagnosis of amyloidosis was established in patients with the following underlying conditions: monoclonal gammopathy of undetermined significance (MGUS) in nine patients (six AL lambda and three AL kappa) and multiple myeloma in seven patients (four AL lambda and three AL kappa).

The medical history of most patients with transthyretin amyloidosis showed that they had restrictive cardiomyopathy (n = 18), severe cardiac arrhythmias (n = 8) and cachexia (n = 3).

In six patients who had clinical signs suggesting infective endocarditis, ATTR amyloidosis was verified by pathological morphology examination.

The patient with AA amyloidosis had familial Mediterranean fever for seven years.

### 2.2. Autopsy Findings

#### 2.2.1. Histology and Immunohistochemistry

The analysis included autopsy samples of 52 deceased persons—28 females (65%) and 24 males (35%) aged from 50 to 96 years, who had systemic amyloidosis. The mean age was 86 years.

Based on the IHC data, ATTR amyloidosis was identified in 21 patients (40%) and AL in 15 patients; AL kappa was seen in 3 (6%) and AL lambda in 12 (23%). A diagnosis of AA amyloidosis was established in 16 cases (31%).

**Figure 3:**
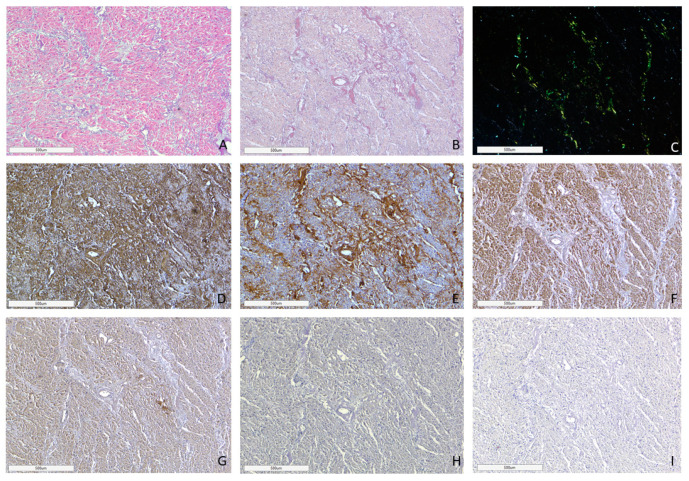
Microscopic image of heart tissue of deceased female patient with AL kappa amyloidosis, 75 years of age. Histological staining with H&E showed deposits of homogenous eosinophilic structures (**A**). Polarized light microscopy of specimens stained with Congo red demonstrated a green birefringence under polarized light, which is specific for amyloid (**B**,**C**). Positive immunostaining with antibodies to amyloid P-component (**D**), anti-κ-light chain antibody (**E**). Negative immunostaining was observed for anti-λ-light chain (**F**), AA amyloid (**G**), anti-TTR (**H**) and anti-LECT-2 (**I**) antibodies. (**A**–**I**) ×40.

AL amyloidosis cases were characterized by a pericellular pattern of amyloid deposition in the myocardial muscle and associated with partial or sometimes complete atrophy of cardiomyocytes (Figure 3). In addition, amyloid masses were detected in subendocardial and subepicardial tissues.

In all cases of AL kappa amyloidosis, both interstitial and intravascular amyloid deposits were present. In the specimens with confirmed AL lambda amyloidosis, both interstitial and intravascular amyloid deposits were detected in 92% of cases, and only interstitial in 8%.

**Figure 4:**
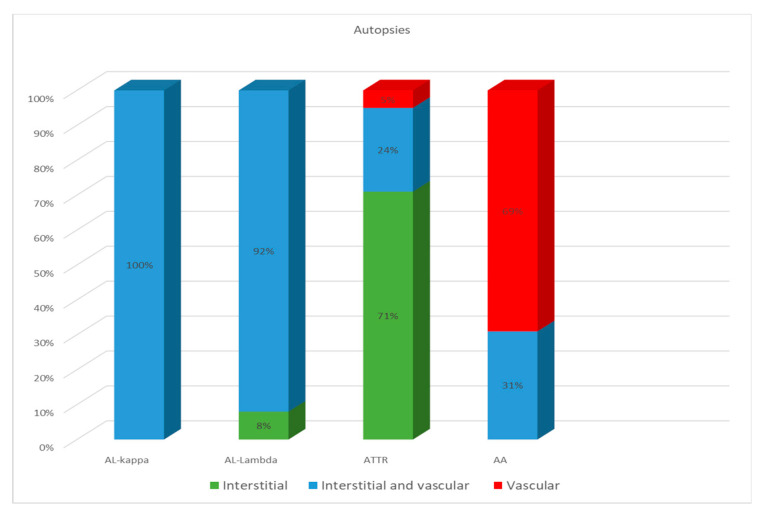
Comparative distribution of amyloid deposits in autopsy specimens of the heart in patients with different types of amyloidosis.

ATTR deposition was identified as interstitial foci in 71% of cases; in 24% both interstitial and vascular tissues were affected, and in 5% of cases, only intravascular deposits were found. In the group of patients with AA amyloidosis, 69% had an intravascular pattern of amyloid deposition, and in 31% of patients, the amyloid masses were present both in the stroma and walls of blood vessels (Figure 4).

**Figure 5:**
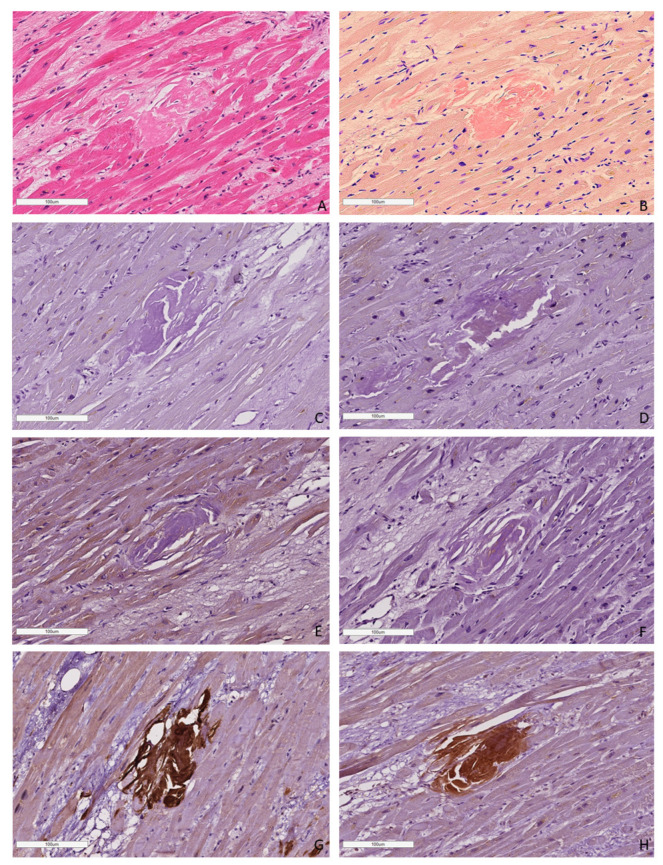
An example of a microscopic image of cardiac tissue of the deceased male patient with ATTR amyloidosis, 82 years of age. Histological staining with H&E showed deposits of homogenous eosinophilic structures (**A**). Staining with Congo red demonstrated bright red amyloid deposits (**B**). Negative immunostaining was observed for AA amyloid (**C**), anti-κ-light chain (**D**), pKLC (**E**) and anti-λ-light chain (**F**) antibodies. Positive immunostaining with the antibodies to amyloid P-component (**G**), anti-TTR antibody (**H**). (**A**–**H**) ×100.

**Figure 6:**
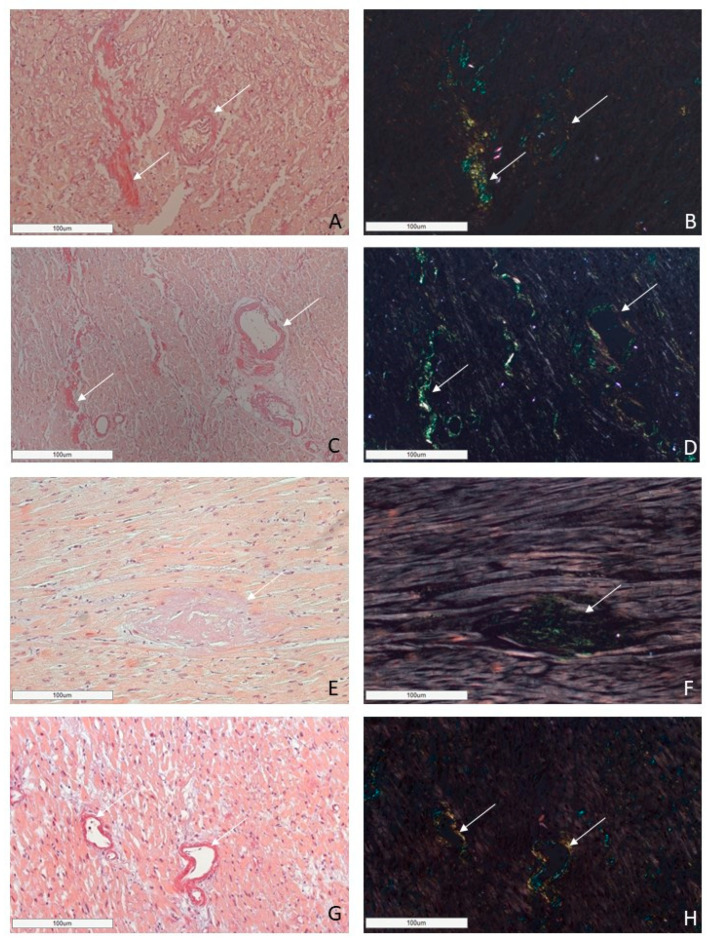
Microscopic images of cardiac tissues in patients with different types of amyloidosis. AL amyloidosis is characterized by diffuse pericellular and intravascular deposition of amyloid displaying apple-green birefringence under polarized light (**A**,**B**—AL kappa amyloidosis; **C**,**D**—AL lambda amyloidosis (white arrow)). Nodular interstitial amyloid deposits in ATTR amyloidosis with typical apple-green birefringence under polarized light (**E**,**F** (white arrow)). AA amyloid deposits within vascular walls, displaying apple-green birefringence under polarized light (**G**,**H** (white arrow)). Staining with CR, ×200.

Based on these findings, we can conclude that the AL type of amyloidosis is often characterized by a diffuse pericellular and intravascular deposition of amyloid, while the ATTR type usually demonstrates focal interstitial dense amyloid deposits. AA amyloidosis in most cases is represented by the intravascular deposition of amyloid masses (Figure 5 and Figure 6).

A correlation analysis of patient age and gender with amyloid type demonstrated that AL amyloidosis was more common in patients during the eighth decade of life (47%), while 48% of cases of ATTR amyloidosis were found in persons during the 9ninth decade of life, and 52% in persons over 90 years of age. Transthyretin amyloidosis was identified more frequently in female patients. AA amyloidosis was found more often in patients in the ninth decade of life (31%), while in the seventh and eighth decades of life, the proportion of patients was the same (25%) (Table 1).

#### 2.2.2. Correlation Analysis of Clinical Data and Post-Mortem Findings of the Group of Autopsy Cases

Clinical diagnosis of amyloidosis in the group of autopsy cases was established in 16 patients (31%), while in other cases, the disease was identified only at the autopsy (69%).

**Figure 7:**
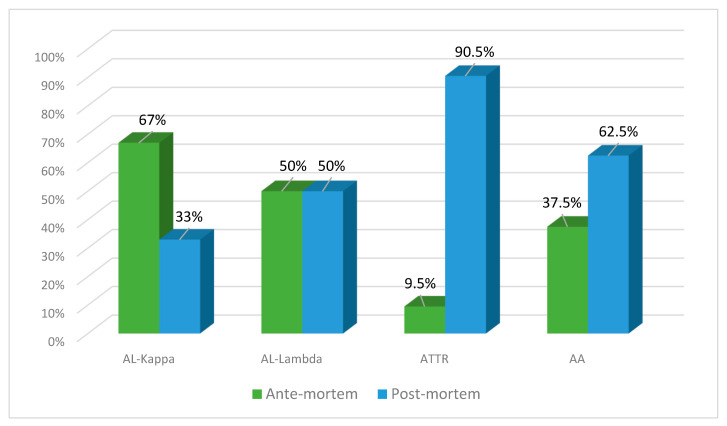
Percentage ratio of ante-mortem (clinical data) and post-mortem (actual findings at the autopsies performed in the same patients) detection of different types of amyloidosis.

The number of ante- and post-mortem-diagnosed cases of AL lambda amyloidosis was equal (50% each), while clinical diagnosis of AL kappa amyloidosis was established in 67% of patients and found at the autopsy in 33% of patients. ATTR amyloidosis was identified at the post-mortem examination in 90.5% of cases and only in 9.5% prior to the patient’s death. The clinical diagnosis of AA amyloidosis was established in 37% of patients, and at the autopsy, it was identified in 62.5% of cases (Figure 7).

In all patients with clinically diagnosed amyloidosis, it was verified in living patients by biopsy findings in the kidneys, duodenum or rectum. In all cases, the diagnosis was confirmed at the autopsy. In patients with AA and AL amyloidosis, the post-mortem examination revealed amyloid deposits not only in the heart muscle, but also in the kidneys, liver, spleen and gastrointestinal system. In ATTR amyloidosis cases, the autopsy results demonstrated the presence of amyloid deposits in the myocardium and pulmonary blood vessels, as well as in the stomach and duodenum walls.

Systemic AL amyloidosis as a primary disease was confirmed in five autopsy cases. In six cases it was concluded that the underlying disease was multiple myeloma with the subsequent development of AL kappa (n = 2) or AL lambda (n = 4) amyloidosis. In four cases of AL amyloidosis, COVID-19 was considered a primary diagnosis.

As a comorbidity, AA amyloidosis was recognized in patients with the following underlying conditions: rheumatoid arthritis with kidney involvement (n = 5); nephrosclerosis (n = 3); novel coronavirus disease (n = 6); familial Mediterranean fever (n = 1); and Still’s disease (n = 1).

ATTR amyloidosis was a comorbidity in patients with such underlying medical conditions as novel coronavirus disease (n = 7), atherosclerotic encephalopathy (n = 4), infectious endocarditis (n = 2), post-infarction cardiosclerosis (n = 5), and cerebrovascular disorders (n = 3).

### 2.3. The Degrees of Amyloid Deposition

**Figure 8:**
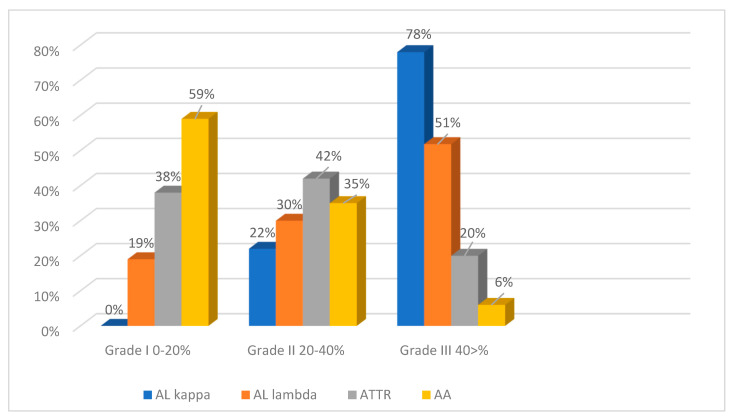
Evaluation of the intensity of amyloid deposits in different types of amyloidosis (EMBs and autopsy specimens).

**Figure 9:**
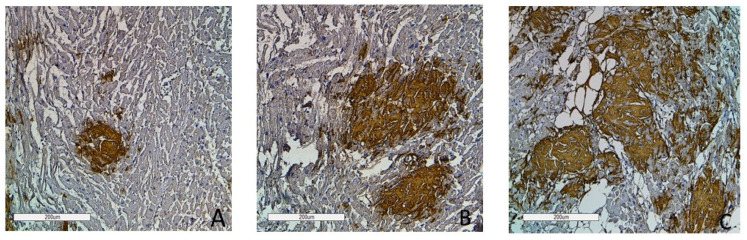
Immunohistochemistry images of amyloid deposition in the myocardium. Grade 1 (0–20% of amyloid deposits) (**A**); Grade 2 (20–40% of amyloid deposits) (**B**); Grade 3 (more than 40% of amyloid deposits) (**C**). Amyloid is stained with polyclonal antibody to Prealbumin, ×100.

To assess the intensity of amyloid deposits in biopsy and autopsy specimens, a grading system was used with three grades of amyloid depositions (Grade 1—0–20% in the field of vision; Grade 2—20–40%; Grade 3—40% and over) based on the clinical significance of the 20% myocardial lesion threshold [2]. The findings demonstrate that the most intense amyloid depositions both in biopsy and autopsy specimens occurred in patients with AL kappa amyloidosis. No cases with Grade 1 amyloid depositions were identified, while Grade 3 depositions were the most common findings (78%). Grade 2 depositions were recorded in 22% of the studied cases. Among the cases of AL lambda amyloidosis, Grade 1 amyloid depositions were found in 19%, Grade 2 in 30% and Grade 3 depositions in 51% of the deceased persons. Most patients with ATTR amyloidosis had amyloid depositions of Grade 2 (42%). Grade 1 was found in 38% and Grade 3 in 20% of cases. In the AA amyloidosis group, the largest proportion of patients had amyloid depositions of Grade 1 (59%), while Grade 2 was identified in 35% and Grade 3 only in 6% of cases (Figure 8 and Figure 9).

## 3. Discussion

So far, endomyocardial biopsy (EMB) remains a gold standard for diagnosing multiple ischemic cardiac disorders, such as viral myocarditis, cardiosclerosis, and infiltrative or restrictive cardiomyopathy, e.g., cardiac amyloidosis [28,29,30]. The indications for EMB include plasma cell dyscrasia in patients with uncertain results of laboratory testing and instrumental methods, as well as when differentiation of AL cardiac amyloidosis from ATTR is required [10].

Though scintigraphy with Technetium-based radiopharmaceuticals (99mTc) is now recognized as the best non-invasive modality for diagnosing ATTR-wt, 20% of patients may have monoclonal gammopathy, which occurs more frequently in AL amyloidosis. In such cases, the differentiation between ATTR-wt and AL amyloidosis is crucial, and EMB with amyloid typing is the only method permitting definitive diagnosis [31].

The grade of amyloid deposition intensity confirmed by endomyocardial biopsy is the key factor for selecting therapeutic management of the disease, especially in patients with AL amyloidosis. As shown, the involvement of more than 20% of the area of an EMB specimen is associated with lower chemotherapy success rates [4]. Thus, increased cardiac involvement in ATTR or AL amyloidosis will be indicative of a worse prognosis [32]. Our study has highlighted the role of EMB as a unique diagnostic procedure for recognizing this important prognostic factor.

Despite the recent advances in non-invasive diagnostics of AL and ATTR amyloidosis, a biopsy procedure is still required in some cases. Though the diagnostic sensitivity of the biopsy of subcutaneous fatty tissue is high (84%) for AL amyloidosis, it is lower for ATTR-wt and ATTR-v amyloidosis (15% and 45%, respectively). Thus, in cases of ATTR amyloidosis, EMB remains the most accurate diagnostic tool for identifying cardiac amyloidosis [33].

In our study, the retrospective analysis of 46 EMBs and 52 autopsies of identified cardiac amyloidosis demonstrated that 83% of patients had either AL or ATTR amyloidosis. A high percentage of AL and ATTR amyloidosis (98%) was determined via EMBs. These findings correlate with the data published earlier by other researchers [34].

It is worth noting that the proportions of AL and ATTR amyloidosis in the autopsy group were lower than those in the group of patients with EMBs. Probably, the difference was underpinned by a higher percentage of AA amyloidosis cases.

The detection of a single case of AA amyloidosis via EMB can be explained by the fact that cardiac involvement in systemic AA amyloidosis is less common than in AL or ATTR amyloidosis. But even if the heart is affected, such patients do not have cardiovascular manifestations, as this type of amyloidosis is usually associated with kidney problems. Since most patients with AA amyloidosis present with rapidly evolving nephrotic syndrome, they should conventionally undergo renal biopsy. Even in those cases of AA amyloidosis where cardiac involvement is suspected, the diagnosis is usually made after renal or rectal biopsy. Therefore, EMB is not recommended for patients with this type of amyloidosis. Thus, the prevalence of cardiac involvement in AA amyloidosis could be higher than detected in EMB specimens. This assumption has been proven in our study, as the diagnosis of AA amyloidosis in 31% of patients was established only at the autopsy.

According to scientific publications, the prevalence of AL amyloidosis is the same in males and females. This trend was also observed in the EMBs examined in our study (men and women were affected equally), but the autopsy reports showed that the ratio of female to male patients was 2:1. Heart failure occurring in patients with AL amyloidosis is associated with extensive amyloid deposits distributed around cardiomyocytes leading to their atrophy, as well as with the direct cytotoxic effect of amyloidogenic immunoglobulin light chains. Amyloidogenic free light chains can impair cardiomyocyte metabolism, causing lysosomal dysfunction, oxidant stress, apoptosis, and the dysregulation of MAP-kinase (MAPK) signaling pathways and autophagy. These data suggest that a direct intracellular cytotoxic effect of amyloidogenic immunoglobulin light chains is also responsible for the rapid progression and poor prognosis of the disease [5].

In our study, ATTR amyloidosis was found more often in the EMBs of male patients (75%), while at the autopsy ATTR amyloidosis was revealed with the same frequency in men and women. Though most of the patients with clinically diagnosed ATTR amyloidosis are men, the percentage of females with this type of amyloidosis is higher in autopsy series [35,36].

A remarkable finding was the postmortem identification of clinically undiagnosed ATTR amyloidosis in seven patients who died from the novel coronavirus disease. Five of these patients had treatment-resistant rapidly progressive heart failure. A microscopic examination of their heart samples revealed multifocal interstitial amyloid deposits. Thus, the fatal outcome was caused by heart failure and comorbid conditions.

Also, the results of our study have demonstrated that ATTR amyloidosis was mostly diagnosed at autopsy (90.5%), suggesting that ATTR may be an underdiagnosed cause of heart failure in elderly patients.

ATTR amyloidosis was found three times more often in the EMBs of male patients than in those of female patients, while at the autopsy, ATTR amyloidosis was detected in men and women almost with the same frequency.

## 4. Materials and Methods

This retrospective observational study was performed using data collected from Petrovsky National Research Centre of Surgery and Savelieva City Clinical Hospital No. 31 from 2013 to 2023. The study was conducted in accordance with the Declaration of Helsinki, and approved by the Institutional Ethics Committee of Avtsyn Research Institute of Human Morphology of Federal State Budgetary Scientific Institution “Petrovsky National Research Centre of Surgery” No. 6, 21 June 2024.

The study included 98 patients with cardiac amyloidosis who were divided into two groups based on the method of examination. The first group consisted of 46 patients with cardiac amyloidosis revealed by endomyocardial biopsies (EMBs), and the second group included 52 amyloidosis patients in whom the morphological diagnosis of cardiac amyloidosis was made at autopsy. Patients from the autopsy group did not undergo EMB prior to death, and the morphological diagnosis of cardiac amyloidosis was established at the autopsy. To collect anamnesis, we used data from medical records, including reports of biopsies from other hospitals where patients were treated.

For performing microscopic examinations, all biopsy and autopsy samples were fixed with 10% neutral buffered formalin solution and embedded in paraffin. Subsequently, paraffin sections with a thickness of 3–5 μm were prepared. Then, hematoxylin and eosin (H&E) and Congo red (CR) staining was performed on all tissue sections. CR staining was performed manually according to the standard protocol [37]. To confirm amyloid deposition, Congo red sections were examined under polarized light. All changes were identified via light and polarized light microscopic examination with polarization blocks (Lieca DM2500, Wetzlar, Germany).

Immunohistochemical (IHC) analysis was performed with the Leica^TM^ BOND-MAX^®^ IHC automatic staining system (Leica, Wetzlar, Germany) using monoclonal Anti-Serum Amyloid P/SAP antibody (1:100, Abcam, Cambridge, UK), polyclonal antibody to Prealbumin (1:100, Cloud-Clone Corp., Katy, TX, USA), monoclonal Anti-Kappa light chain antibody (1:200, Abcam, Cambridge, UK), monoclonal Anti-Lambda light chain antibody (1:200, Abcam, Cambridge, UK), and monoclonal anti-AA amyloid (1:100, Clone C3, Cloud-Clone Corp., Katy, TX, USA).

The IHC method was used to detect the presence of all amyloid types based on positive staining results with the antibody to amyloid P-component (monoclonal Anti-Serum Amyloid P/SAP antibody). ATTR amyloidosis was diagnosed by positive IHC staining results with the antibodies to TTR (polyclonal antibody to Prealbumin); AL kappa and AL lambda types were determined via positive IHC staining results with the antibodies to κ- light chain (monoclonal anti-Kappa light chain antibody) and λ-light chain (monoclonal anti-Lambda light chain antibody), respectively, and AA amyloidosis was determined via positive IHC staining results with the antibodies to AA amyloid (monoclonal anti-AA amyloid).

To assess the intensity of amyloid deposits, a semi-quantitative grading system was used with three grades of amyloid depositions, as follows: Grade 1—amyloid deposition made up less than 20% in the field of vision; Grade 2—20–40%; Grade 3—40% and over.

We used these grading criteria to assess the extent of cardiac involvement. This factor could play an important role in clinical practice, as the effectiveness of the administered therapy may decrease in cases in which amyloid deposition comprises more than 20% in the field of vision [2].

## 5. Conclusions

Our study has demonstrated that the clinical diagnosis of cardiac amyloidosis is challenging. The fact that patients with ATTR commonly remain undiagnosed prior to death raises the issue of expanding indications for EMB. Another important conclusion is associated with the intensity of amyloid deposition. As shown, the most intense amyloid deposits both in the examined EMBs and in the autopsy reports were revealed in patients with AL kappa amyloidosis, suggesting that the disease has a more aggressive and quickly progressing clinical course, leading to a poorer prognosis for patients with this type of AL amyloidosis.

## Figures and Tables

**Table 1:** Correlation of amyloidosis types in EMBs and autopsy specimens with age and gender of the patients.

Age, n (%)															Gender, n (%)			
Type of Amyloidosis	Patients, n (%)		40–49		50–59		60–69		70–79		80–89		90–99		Women		Men	
B/A *	B	A	B	A	B	A	B	A	B	A	B	A	B	A	B	A	B	A
AL kappa	6 (13)	3 (6)	-	-	1 (16)	1 (33)	1 (16)	1 (33)	3 (52)	1 (33)	1 (16)	-	-	-	4 (67)	2 (66)	2 (33)	1 (33)
AL lambda	15 (33)	12 (23)	2 (13)	-	3 (20)	2 (16)	3 (20)	4 (34)	7 (47)	6 (50)	-	-	-	-	7 (45)	8 (66)	8 (55)	4 (34)
ATTR	24 (52)	21 (40)	-	-	3 (13)	-	-	-	13 (54)	-	7 (30)	10 (48)	1 (3)	11 (52)	6 (25)	10 (48)	18 (75)	11 (52)
AA amyloidosis	1 (2)	16 (31)	-	-	-	3 (19)	1 (100)	4 (25)	-	4 (25)	-	5 (31)	-	-	1 (100)	8 (50)	-	8 (50)
Total, n (%)	46 (100)	52 (100)	2 (4)	-	7 (15)	6 (11)	5 (12)	9 (17)	23 (50)	11 (21)	8 (18)	15 (29)	1 (2)	11 (21)	18 (39)	28 (54)	28 (61)	24 (46)

Biopsy/Autopsy *.

## Data Availability

All data and materials are available upon reasonable request. Address to Z.V.G. (email: gioeva_z@mail.ru) or L.M.M. (email: mikhalevalm@yandex.ru), Avtsyn Research Institute Of Human Morphology of Federal State Budgetary Scientific Institution “Petrovsky National Research Centre of Surgery”, Moscow, Russia.

## Abbreviations

AA, amyloid A protein; AL, amyloid light chain; ATTR, amyloid transthyretin; COVID-19, Coronavirus-19 disease; CR, Congo red; H&E, hematoxylin plus eosin; IHC, immunohistochemistry.
